# The Ward Locker Competition: The Successful Designs

**Published:** 1914-05-30

**Authors:** 


### THE PRACTICAL EQUIPMENT OF A HOSPITAL.

### THE WARD LOCKER COMPETITION.

### The Successful Designs.

This competition has had considerable practical value. The lockers (for a full description of each see The Hospital, May 9, pp. 161-4) collectively represent sound inside knowledge, keen intelligence applied to the special requirements of individual hospitals, marked ability, and simplicity and thoroughness in the letterpress descriptions, especially of Lockers F and A, as well as courage in criticising the defects of several of the selected designs on the part of those who submitted them for the competition. Our thanks are due to all the competitors, and we hope that equal practical knowledge and thoroughness may be exhibited in future competitions in connection with this practical section of the paper. There is no doubt that:—

Locker F combines greater advantages and the maximum of uses. Those responsible for the design and construction of this locker are entitled to great credit for the ingenuity and efficiency displayed.

Locker A comes next, which for a small and cheap locker, carefully thought out and admirably constructed, offers many advantages.

Locker D is entitled to praise for use in an institution where the fullest hygienic precautions are essentials in the equipment. We believe, however, that after it has been in use at consumption and other contagious hospitals, like Locker B the design will probably be modified in certain directions.

Locker E for a small infirmary and hygienically is worthy of commendation.

Locker C is simple and somewhat primitive, but we expect it will be found popular in a sanatorium. It has the merit of cheapness, and the fact that American willow is used in its construction is an advantage, because the wood is hard and very durable, as well as inexpensive. It is wonderfully cheap at 19s.

We may further usefully point out that *pine is not a suitable wood in any circumstances for lockers*; if wood is used it should be either teak or oak or American willow, or some other hard wood which has proved satisfactory after experience of the wear and tear to which it is subject in a busy hospital ward.

N.B.—We shall be glad to have an estimate of the cost of Locker F; also of Lockers B and E.

#### Names of the Prize-Winners.

After a full consideration and examination of all the voting papers sent in, and in view of the evident attempts to influence the voting which we have already referred to, we award:

**The First Prize of Five Guineas to Locker F,** which was submitted by J. T. Rudd, Esq., Secretary of the Coventry and Warwickshire Hospital, Coventry.

**The Second Prize of Two Guineas to Locker A,** submitted by Miss Davies, matron of the Grimsby and District Hospital, Grimsby.

#### The Special Prize.

No voting paper was arranged in accordance with the majority of opinions expressed by the voters, and the Editor has accordingly decided in his discretion to divide the Special Prize of Three Guineas as follows:—

One guinea to Miss Anna Sinclair, matron, Great Barr Park Hospital, near Birmingham, who submitted the design for Locker D.

One guinea to Miss M. Lillie, matron, North Devon Infirmary, Barnstaple, who submitted the design for Locker E.

Half a guinea each to Frank G. Hazell, Esq., Secretary, Norfolk and Norwich Hospital, Norwich, and to Miss Helen M. Brown, Daneswood Sanatorium, Woburn Sands, who respectively submitted the designs for Lockers B and C.

